# Milk and Whole Blood Surveillance Following Lethal and Sublethal Lead Intoxication in a Michigan Dairy Herd

**DOI:** 10.3390/toxics13060445

**Published:** 2025-05-28

**Authors:** Rachel Sheffler, Sarah Rebolloso, Isaiah Scott, John P. Buchweitz, Birgit Puschner

**Affiliations:** 1 Veterinary Diagnostic Laboratory, College of Veterinary Medicine, Michigan State University, Lansing, MI 48910, USA; sheffle5@msu.edu (R.S.);; 2 Department of Pathobiology and Diagnostic Investigation, College of Veterinary Medicine, Michigan State University, Lansing, MI 48910, USA; 3 Michigan Department of Agriculture and Rural Development, Lansing, MI 48933, USA

**Keywords:** lead, toxicosis, bovine, lead contamination, residue, human health risk, food safety, milk

## Abstract

Lead contamination in the environment affects both humans and animals. Even with the decrease in manufactured items containing lead, contaminants persist in the landscape and may enter the food supply through animal products. In cattle, lead poisoning is associated with economic losses due to mortality and treatment costs and poses a health risk to consumers. A dairy herd was exposed to lead through feed that was contaminated with a 12-volt battery from a mixer wagon. Lead concentrations in blood and milk samples were examined over 289 days. A 2 ng/mL threshold for lead in milk was utilized to release affected cows back into the milking herd. After 289 days of surveillance, one of the five cows under milk surveillance was yet to meet this threshold. Milk lead concentrations greater than 2 ng/mL can result in lead intakes exceeding 2.2 µg/day limits for young children in the highest milk consumption group. Lead is not routinely assessed in fluid milk as a quality control step prior to processing in the United States, yet interstate commerce justifies a need for harmonized protocols for routine lead surveillance of the general milk supply and enhanced surveillance and quarantine for known food-animal exposures.

## 1. Introduction

Producers and veterinarians play a critical role in ensuring a safe, healthy, and nutritious food supply that is free from contaminants, such as lead, in fluid milk and dairy products. Milk consumption in young children (<2 years) and primary-school-aged children (4–11 years) provides essential nutrients such as calcium, iodine, riboflavin, magnesium, potassium, vitamins, and protein [1,2,3]. Since children consume more milk than adults on average and are more vulnerable to the toxic effects of lead, safeguarding the milk supply following non-lethal lead exposures in dairy cattle through enhanced surveillance efforts is a key step in the management of bovine lead intoxications.

Lead exposure gained national media attention in Michigan during the Flint water crisis [4]. This public health emergency led to increased research and data on the health effects and educational proficiencies in young children exposed to lead, as well as heightened awareness and consumer demands for a safe water and food supply. Lead exposure in young children and mother-to-child transmission via the placenta are linked to direct neurotoxic effects [5]. Early childhood exposures are associated with increased anxiety and behavioral problems and decreased brain volume, executive functioning, and academic achievement [4,5].

Diverse sources of lead in the environment continue to cause acute and chronic intoxications in companion and production animals and wildlife species [6]. Reports of intoxications in Holstein herds report anthropogenic source materials such as lead shot, wood ash, paint, and lead acid batteries to be causative [7]. Because of the potential transfer of lead into animal-derived products, such as milk, meat, and eggs, it is particularly important to perform thorough investigations of any suspected lead poisonings in food animals. This includes identification and removal of source materials and isolating subclinical animals that may still harbor potentially harmful concentrations of lead. Lead toxicosis in food animals is not a reportable disease in all states across the USA. As such, no federal or state action levels for lead in fluid milk have been universally established nor adopted. Acceptable limits, quarantine protocols, and surveillance efforts are determined on an individual basis by the state regulatory officials. With a blood half-life of 1–2 months, elevated blood lead concentrations are indicative of recent exposure and are used for monitoring of nonlethal lead intoxication and decision-making in quarantined herds [8,9]. Here we present a case of acute lead intoxication in a dairy cow herd and the subsequent surveillance protocols utilized to monitor blood and milk lead concentrations prior to releasing animals back into the milking herd. The objective of this study is to demonstrate milk surveillance as a less invasive and more direct strategy to assess safety of the milk supply following sublethal lead exposure events in lactating and soon-to-be lactating dairy cattle. We hypothesize that blood and milk lead concentrations will decrease together throughout a several months long quarantine period following sublethal exposure.

## 2. Materials and Methods

### 2.1. Initial Investigation and Workup

On 24 April 2024 (day 0 of exposure), the producer noted decreased ruminations in the dry cow herd of 28 animals. On day 2, the herd veterinarian was contacted to discuss the case. Several deaths were noted in the affected group and the incident was reported to the Michigan Department of Agriculture and Rural Development (MDARD) Animal Industry Division out of concern for lead toxicosis. Lead intoxication is a reportable disease in the state of Michigan. The herd was placed under a verbal quarantine. MDARD conducted an initial investigation on day 5 following the report and began with interviews of the producer, herd veterinarian, and a facility inspection. Blood samples were collected from all cows in the dry cow herd for lead analysis. One cow had calved since the exposure; a blood sample was collected from the cow–calf pair and a milk sample was collected. Milk from this cow was discarded and not incorporated into the bulk tank. Additional bulk tank milk samples were collected for assessment by MDARD Dairy staff and analyzed by the Michigan State University Veterinary Diagnostic Laboratory (MSU VDL).

Testing intervals were based on calving and freshening of the dry cow group. Planned blood samples were collected within 1–2 days following calving. Paired cow–calf blood samples and a milk sample were collected as each cow freshened. Blood and milk samples were also collected from any exposed cows that freshened prior to that collection date. The final calf was born at the end of June 2024. After June calving, the sampling interval was extended to 3-month intervals.

### 2.2. Laboratory Lead Analysis

Whole blood and milk samples were diluted 25-fold with a solution containing 0.5% Ethylenediaminetetraacetic acid (EDTA) (Sigma Aldrich, St. Louis, MO, USA) and Triton X-100 (Sigma Aldrich, St. Louis, MO, USA), 1% ammonium hydroxide (Thermo Fisher Scientific, Waltham, MA, USA), 2% 1-butanol (Sigma Aldrich, St. Louis, MO, USA), and 5 ppb of scandium and 7.5 ppb of germanium, rhodium, indium, and bismuth as internal standards. Lead analysis utilized a previously published method using an Agilent 7900 Inductively Coupled Plasma—Mass Spectrometer (ICP/MS) (Agilent Technologies Inc., Santa Clara, CA, USA) The ICP/MS was tuned to yield a minimum of 7500 cps sensitivity for 1 ppb yttrium (mass 89), less than 1.0% oxide level as determined by the 156/140 mass ratio and less than 2.0% double charged ions as determined by the 70/140 mass ratio [10]. Elemental concentrations were calibrated using a 6-point linear curve of the analyte–internal standard response ratio. Standards were sourced from Inorganic Ventures (Christiansburg, VA, USA). A second source calibration check standard from High Purity Standards (Charleston, SC, USA) was also used. Lypochek (Bio-Rad, Hercules, CA, USA) heavy metal standards were used as controls.

### 2.3. Diagnostic Criteria and Decision Making

According to the MDARD standard operating procedure for lead toxicity/exposure response (rev. 5 October 2012), an animal may be released from quarantine once a single blood lead measurement is ≤100 ng/mL or when two consecutive blood lead measurements, are between 100 and 200 ng/mL with no known continued exposure and no clinical signs of illness [11]. The Dairy, Feed and Produce Division, in consultation with the FDA Center for Food Safety and Applied Nutrition (CFSAN) and MDARD toxicologist, determined that for an individual cow to be reintroduced to the milking herd and its milk incorporated into the bulk tank, its milk lead concentration must be less than 2 ng/mL.

### 2.4. Statistics and Calculations

Data were analyzed and plotted using GraphPad Prism software (GraphPad Prism 10.4.1, Boston, MA, USA). Statistical analysis was performed using RStudio (Posit RStudio 2025.05.0+496, Boston, MA, USA). The analysis applied a linear mixed-effects model to assess the relationship between milk Pb levels (Milk) and two explanatory variables: Day of sample collect and Blood Pb levels [12,13,14,15,16,17,18]. To address skewness in the milk yield data, a log transformation (log(Milk Pb + 1)) was applied to the response variable.

To account for repeated measurements from the same cows, a random intercept for Cow_ID was included in the model. The model was fitted using the lmer() function from the lmerTest package, which provides the *p*-value for fixed effects using Satterthwaite’s method for degrees of freedom approximation [13].

The model’s assumptions were evaluated through diagnostic plots using the check_model() function from the performance and see packages [15,17]. Additionally, the random effects structure was visualized using a dotplot, and predicted vs. observed values were plotted to assess model fit [16]. The response variable was log-transformed; the model coefficients can be interpreted as approximate percent changes in milk yield for a one-unit change in the predictor.

## 3. Results

### Case Progression

On 24 April 2024 (day 0), all 28 cows in the dry pen were noted to have decreased ruminations after a new pile of beet pulp was added to the total mixed ration. Two days later, 26 April (day 2), the herd veterinarian was contacted to clinically assess the herd. By day 3, 13 deaths occurred prompting lead analysis of 4 whole blood and 1 bulk tank milk samples. Blood lead concentrations in the four submitted cows were 7, 699, 509, and 1055 ng/mL with 3 of 4 cows exceeding the 350 ng/mL threshold for acute toxicosis [8]. Cow Q had recently calved and returned to the milking herd prior to the onset of clinical signs in the dry pen and was not included in the exposure group. The lead concentration in the fluid milk from the bulk tank was not detected for lead. In Michigan, any “toxic substance contamination” must be reported within 24 h, and MDARD was contacted accordingly. Following confirmation of elevated lead concentrations, the farm was quarantined and milk from affected cattle was prohibited from entering the bulk tank as surviving cows calved and freshened.

**Figure 1:**
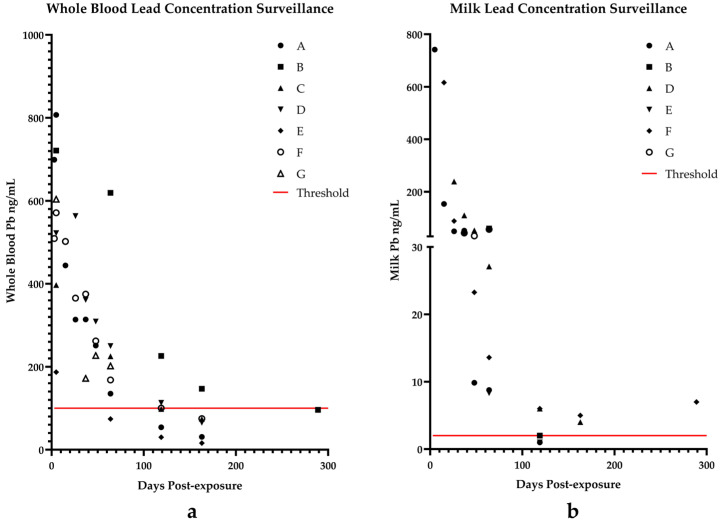
Decreasing trends of whole blood (**a**) and milk (**b**) lead concentrations across 289 days of post-exposure surveillance for cows A–G. Red lines designate regulatory threshold values required for release from quarantine.

During the investigation on 29 April (day 5), the owner reported finding pieces of a battery in the ration that was incorporated into the mixer wagon following the addition of a bale of hay. By the time field inspectors arrived, most feed had been consumed by the animals and any remaining feed had been spread on a field. The affected feed was intended only for the dry cow pen and was not reported to be fed to any of the lactating cattle. On physical examination, dry cows were found in various conditions ranging from clinically normal to dull and listless. One animal experienced blindness, and two others were recumbent and unable to rise. Cattle in the milking herd were reported as bright, alert, and responsive. Blood samples were collected from 14 affected dry cows and blood and milk samples were collected from one cow and her calf that was born since the exposure event. Whole blood concentrations ranged from 187 to 2587 ng/mL with 14/15 cows. The cow that calved had a blood lead concentration of 807 ng/mL and a milk lead concentration of 742 ng/mL; the whole blood lead concentration of her calf was 16 ng/mL. Between 29 April and 13 May (days 5–19 post exposure), 8 of the 15 cows died. The remaining seven cows were monitored through 10 July (day 77) when one additional cow died due to post-calving complications. Blood lead concentrations were monitored in four additional calves; one calf was stillborn, and data is not available. Data for whole blood and milk concentrations are presented in Table 1 and are illustrated in Figure 1a. Cows were released from quarantine once blood lead concentrations were below 100 ng/mL. On 27 June (day 64), a requirement that individual cow milk lead concentrations must test below 2 ng/mL prior to inclusion of their milk in the bulk tank was established. Milk lead concentrations continued to be monitored in 6 affected cows through 7 February 2025 (day 289) and are shown in Table 2 and Figure 1b.

At day 119, three cows had individual milk lead concentrations at or below the 2 ng/mL regulatory threshold for inclusion in the bulk tank. On day 163, two cows had milk lead concentrations exceeding the 2 ng/mL limit. Cow D was sold prior to milk sampling on day 289 and cow F continued to exceed the 2 ng/mL limit and was yet to be released into the milking herd.

**Figure 2:**
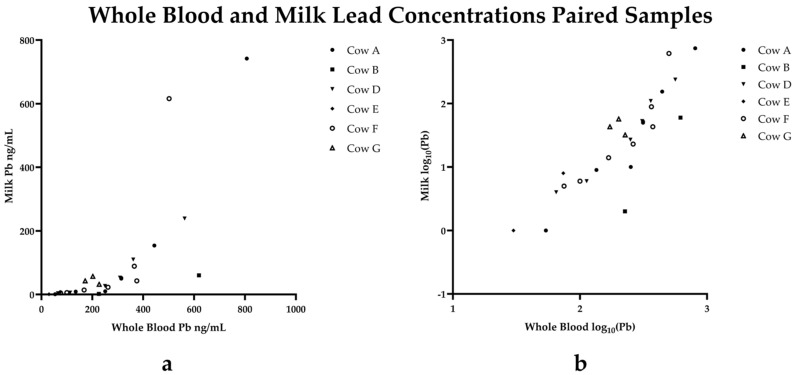
Paired whole blood and milk lead concentrations (**a**) and following logarithmic transformation of data (**b**).

Paired milk and blood samples were analyzed by mixed effects linear regression analysis and a scatterplot displaying paired milk versus lead concentrations with and without logarithmic transformation are presented (Figure 2). Repeated measures were accounted for by including the cow identifier as a random effect within the model. The results of the linear mixed model are presented in Table 3. Both days since exposure and blood lead concentration were significantly associated with the log(milk lead levels). The estimated coefficient for days since exposure was approximately −0.012. This means that for every additional day following the initial lead exposure, the expected log-transformed milk lead decreased by 0.012 units when holding blood constant. This corresponds to a ~1.2% decrease in milk lead per extra day following exposure on average. The coefficient for blood lead concentration was approximately 0.0057. This indicates that for each one-unit increase in the blood lead concentration, the log-transformed milk lead concentration increased by 0.0057. Translating this to original units, milk lead concentrations increased by about 0.57% per unit increase in blood lead concentration.

## 4. Discussion

Lead exposures in cattle are not uncommon and are routinely diagnosed at veterinary diagnostic laboratories across the United States (USA) and across the world and pose a considerable health risk to consumers [19]. Despite this, lead toxicosis in food animals is not a reportable disease in all states across the USA and federal or state action levels for lead in fluid milk do not exist. Rather, in production settings, acceptable limits, quarantine, and surveillance efforts are determined on an individual basis by the state regulatory officials. With a blood half-life of 1–2 months, elevated blood lead concentrations are indicative of recent exposure and are used for monitoring of nonlethal lead intoxication and decision-making in quarantined herds [8,9].

The presented case details a reportable lead exposure incident that resulted in lethal lead intoxication in twenty-one animals and sublethal clinical intoxication in an additional seven dry cows. Of the seven dry cows, five calves were born and survived of which three had blood lead concentrations greater than 100 ng/mL. One calf was stillborn and the cow dried-off and a second was born alive but died shortly after birth before a blood sample could be collected for lead analysis. Lead toxicosis could have played a role in the stillbirth and calf death; however, supporting data are not available to demonstrate a cause-and-effect relationship or rule out infectious causes of stillbirth. Calves were not followed for additional surveillance or health monitoring following their release from quarantine. Data are not available to assess the impact of in-utero lead exposure on calf growth and development following birth. Future prospective studies of lead exposed calves would provide critical data for the assessment of long-term health and production outcomes in affected cattle.

Following the initial exposure and resulting deaths, attention shifted to determining the fate of the surviving animals. Once quarantine was placed, options for the producer included (1) euthanasia and disposal of animals exceeding lead action levels, (2) continued quarantine and surveillance until blood lead concentrations were below 100 ng/mL at which time animals would be sent for slaughter, or (3) continued quarantine and surveillance until animals were released from quarantine by the state and milk was deemed safe to enter commerce. Previous reports of long elimination half-lives of lead in dairy cows and prolonged elevations of blood lead concentrations months to years after exposure result in long quarantines that can place further economic strain on dairy farmers [7,8,9,20,21,22]. In the reported case, the producer agreed to quarantine and continued lead surveillance to determine whether individual quarantines could be lifted.

When assessing blood lead alone, one cow was released from quarantine after 64 days, two cows after 119 days, two cows after 163 days, and one cow after 289 days. Three cows were released from milk holds after 119 days whereas cows D and F were yet to be released from milk surveillance. Rather than analyzing paired milk and blood samples from each cow throughout quarantine, monitoring milk lead concentrations in lactating animals can provide a non-invasive sampling method and provide estimations of circulating lead concentrations. Here, we found a positive correlation between blood and milk lead concentrations, which is consistent with previous reports of a strong positive correlation between blood and milk lead concentrations using paired samples collected early in lactation in a group of sub-clinically exposed dairy cattle [20]. From a food safety perspective, testing milk lead concentrations directly rather than monitoring whole blood lead of affected lactating cattle provides a non-invasive approach and generates the most relevant information for risk assessment and consumer safety evaluation.

Since milk is largely consumed by infants and children, lead residues in milk are of particular concern, especially in children where even low-level exposure in early childhood is known to cause cognitive development deficits [4,5]. Considering the most susceptible population, young children consuming cow’s milk, Michigan state regulatory officials determined that individual cow milk samples cannot exceed 2 ng/mL for release from milk surveillance testing and re-introduction into the milking herd. This risk assessment was based on FDA-issued guidance and an interim reference level of 2.2 µg/day of lead for young children [23]. Based on the 90th percentile of milk consumption in children ages 0 to 6 derived from the National Health and Nutrition Examination Survey (NHANES) available through the FDA Center for Food Safety and Applied Nutrition (CFSAN), a milk intake of 41.70 g/kg body weight per day was utilized for risk assessment purposes for an average 6-year-old child weighing 31.8 kg [24]. Assuming an average 6-year-old at the highest intake level consumes 1326 mL of milk, fluid milk should not contain greater than 1.7 ng/mL lead to not exceed the 2.2 µg/day limit. This is further emphasized in an FDA total diet study which found that while milk and yogurt contained relatively low lead concentrations (less than 5 ng/g), they remain an important source of lead exposure in young children due to higher volumes consumed compared to other products with higher lead concentrations but eaten intermittently or in very low quantities [25]. The FDA Total Diet Study data estimated intake of dairy-based foods for children 1–6 to be 428 g per day [25]. Assuming all 428 g of dairy product to be fluid milk, repeating the above calculations results in a limit of 5 ng/mL lead in milk; however, this could result in children in the highest consumption groups exceeding the 2.2 µg lead per day maximum intake.

Of the five cows still producing milk at 119 days of quarantine, only three were released from milk holds and could be returned to the milking herd. After 289 days, one cow had a milk lead concentration exceeding the 2 ng/mL action limit set by the state regulatory agency. This is consistent with previous reports that showed a single cow milk lead concentration greater than 2 ng/mL 800 days after an acute lead exposure [20]. Of note, the cows described in the study by Bischoff et al. had peak lead concentration intervals of 126–306 days which may have resulted from changes in bone resorption and lead release from bone depots during various stages of gestation and lactation [20]. While there is concern that cows that freshen within seven months of sublethal lead toxicosis may develop clinical lead toxicosis with mobilization of bone, there is greater concern over the sub-clinical effects of lead exposure in dairy cows and the potential health risk to humans. Cows with sub-clinical lead exposure do not present with clinical signs suggestive of lead poisoning but can excrete lead into milk at concentrations exceeding the milk action limit for this case with subsequent incorporation into the bulk tank milk [8].

Lactating cows in the affected group were subject to milk lead surveillance until milk lead residues fell below the 2 ng/mL cut-off. This value is ten times lower than current recommendations for fluid milk in the EU [26]. It is of note that prior to instituting the 2 ng/mL action limit in affected cows, the bulk tank lead concentration was 4 ng/mL from the unaffected milking herd. Currently, there are limited data available for background milk lead concentrations in cows not under surveillance or quarantine following an acute exposure event in the USA. A meta-analysis including 72 studies assessing milk metal concentrations found that 203 raw milk samples from commercial sources contained average lead concentrations of 93 ng/mL (range 4–228 ng/mL) compared to an oral minimum risk level of 41 ng/mL [27]. Across the globe, lead contamination in cow’s milk results in significant lead exposures particularly in highly contaminated areas of developing countries such as Pakistan (23,240 ± 300 ng/mL), Egypt (4400 ± 1600 ng/mL), and India (60,000 ng/mL) whereas developed countries such as the Republic of South Africa (<2 ng/mL), Canada (<2 ng/mL), and Spain (3 ± 0.6 ng/mL) have lower milk lead contamination [27,28] Further, data generated from dairy cattle in the United Kingdom found milk lead concentrations averaging 4.82 ng/mL [range 0.02–15.72 ng/mL] [29]. These data illustrate that lead contamination in milk can serve as a significant source of lead exposure, particularly in young children across the globe.

Interstate commerce and transportation across state lines justifies a need for harmonized protocols for routine lead surveillance of the general milk supply and enhanced surveillance and quarantine for known food-animal exposures. Lead is not routinely assessed in fluid milk as a quality control step prior to processing in the USA. The Code of Federal Regulations (CFR) details standards for milk and cream products but does not specify specific requirements for lead content in fluid milk, cream, or milk products [30]. Moreover, the Codex Alimentarius states that exposed dairy animals with elevated lead concentrations should not be used as a source of milk until lead decreases below action levels set by national authorities [31]. The European Union (EU) set maximum thresholds of 20 ng/mL lead in fluid milk and infant formulas and 100 ng/g in edible bovine tissues [26]. Lead assessment in young children is a clear public health concern; in 2024 Michigan launched updates to the Public Health Code requiring blood lead testing for all children at ages 12 and 24 months. Co-surveillance of milk lead and human blood lead concentrations should be undertaken to provide crucial data to analyze trends in lead burden and milk consumption. This critical data will assist veterinarians and public health officials to establish harmonized state and federal regulatory guidance to ensure the safety of the milk supply while minimizing the economic impact of lead contamination for dairy producers.

## 5. Conclusions

Lead can cause developmental and cognitive deficits even at very low concentrations and any exposure, particularly in young children, is considered potentially harmful. Contamination of milk with lead in non-lethal or sub-clinical exposures in dairy cattle can result in significant lead exposures in children. A lack of federal action levels and milk lead surveillance strategies may result in significant and unnecessary lead exposures. Harmonized guidance and surveillance efforts will mitigate lead exposures through fluid milk and milk products and are necessary to ensure a safe and nutritious food supply.

## Figures and Tables

**Table 1:** Whole blood lead concentrations in affected cows and their calves by days since exposure.

	Days Since Exposure											
Animal ID	3	5	7	15	26	37	48	64	65	119	163	289
A	699	807		444	314	314	251	135		54	31	
B		721						619		226	147	96
C ***		397						225		98	74	
D †		522			563	361	309	250		113	65	
E		187						74		30	16	
F	509	571		502	365	375	262	168		100	75	
G **		604				172	227	202				
H *		2039										
I *		1180										
J *		869										
K *		579										
L *		2587										
M *		930										
N *		745										
O *		735										
P *	1055											
Q ‡	7											
A-Calf			16									
B-Calf									114		60	
E-Calf								27				
F-Calf					198	91						
G-Calf						112	98					

All values are reported in ng/mL (ppb). * Cows H-P died between days 3–19 post-exposure. ** Cow G euthanized on day 77 due to a displaced abomasum following calving. *** Cow C had a stillbirth and was dried off; milk and calf blood lead concentrations are unavailable. † The calf from Cow D died before a blood sample was taken and blood lead is not available. Cow D was sold prior to day 289. ‡ Cow Q calved before the exposure event and was not exposed to contaminated feed. Grey boxes indicate that cows died or were removed from the herd at the specified timepoints and were not available for sampling.

**Table 2:** Milk lead concentrations (ng/mL) in surviving cows following freshening by days since exposure.

	Days Since Exposure								
Animal ID	5	15	26	37	48	64	119	163	289
A	742	154	50	52	10	9	<1		
B						60	2		
D *			239	110	53	27	6	4	
E						8	1		
F		616	89	43	23	14	6	5	7
G **				43	32	57			

* Cow D was sold prior to day 289 milk sampling. ** Cow G euthanized on day 77 due to a displaced abomasum following calving. Grey boxes indicate that cows died or were removed from the herd at the specified timepoints and were not available for sampling.

**Table 3:** Estimated regression parameters, standard errors, degrees of freedom, t-values, and *p*-values for the linear mixed effect model.

	Estimate	Std Error	df	t-Value	*p*-Value
Intercept	2.49	0.51	19.46	4.87	0.0001
Days since exposure	−0.012	0.0035	22.35	−3.49	0.0021
Blood lead	0.0057	0.00084	22.98	6.72	<0.0001

## Data Availability

Data from this study are available from the Michigan Department of Agriculture upon reasonable request via a Freedom of Information Act (FOIA) request.

## Abbreviations

The following abbreviations are used in this manuscript:

MDARD	Michigan Department of Agriculture and Rural Development
MSU VDL	Michigan State University Veterinary Diagnostic Laboratory
NHANES	National Health and Nutrition Examination Survey
CFSAN	FDA Center for Food Safety and Applied Nutrition
FOIA	Freedom of Information Act
CFR	Code of Federal Regulations
EDTA	Ethylenediaminetetraacetic acid
ICP-MS	Inductively Coupled Plasma Mass Spectrometry
EU	European Union
USA	United States of America
